# Insights into the Stability of Graphene Oxide Aqueous Dispersions

**DOI:** 10.3390/nano12244489

**Published:** 2022-12-19

**Authors:** Codrut Costinas, Catalin Alexandru Salagean, Liviu Cosmin Cotet, Monica Baia, Milica Todea, Klara Magyari, Lucian Baia

**Affiliations:** 1 Faculty of Physics, Babeș-Bolyai University, M. Kogălniceanu 1, RO-400084 Cluj-Napoca, Romania; 2 Laboratory for Advanced Materials and Applied Technologies, Institute for Research, Development and Innovation in Applied Natural Sciences, Babeș-Bolyai University, Fântânele 30, RO-400294 Cluj-Napoca, Romania; 3 Faculty of Chemistry and Chemical Engineering, Babeș-Bolyai University, Arany Janos 11, RO-400028 Cluj-Napoca, Romania; 4 Nanostructured Materials and Bio-Nano-Interfaces Centre, Interdisciplinary Research Institute on Bio-Nano-Sciences, Babeș-Bolyai University, Treboniu Laurian 42, RO-400271 Cluj-Napoca, Romania; 5 Faculty of Medicine, Iuliu Hațieganu University of Medicine and Pharmacy, Victor Babeș 8, RO-400012 Cluj-Napoca, Romania; 6 Department of Applied and Environmental Chemistry, University of Szeged, Rerrich B. Sqr. 1, 6720 Szeged, Hungary

**Keywords:** graphene oxide, stability, sono-chemical exfoliation, structural characterisation, aqueous dispersions

## Abstract

Understanding graphene oxide’s stability (or lack thereof) in liquid solvents is critical for fine-tuning the material’s characteristics and its potential involvement in future applications. In this work, through the use of structural and surface investigations, the alteration of the structural and edge-surface properties of 2D graphene oxide nanosheets was monitored over a period of eight weeks by involving DLS, zeta potential, XRD, XPS, Raman and FT-IR spectroscopy techniques. The samples were synthesized as an aqueous suspension by an original modified Marcano-Tour method centred on the sono-chemical exfoliation of graphite. Based on the acquired experimental results and the available literature, a phenomenological explanation of the two underlying mechanisms responsible for the meta-stability of graphene oxide aqueous dispersions is proposed. It is based on the cleavage of the carbon bonds in the first 3–4 weeks, while the bonding of oxygen functional groups on the carbon lattice occurs, and the transformation of epoxide and hydroxyl groups into adsorbed water molecules in a process driven by the availability of hydrogen in graphene oxide nanosheets.

## 1. Introduction

In 2004, the isolation of free-standing graphene provided the groundwork for 2D material research [1]. Many 2D materials have been separated since then, and graphene has been at the forefront of many research efforts [2]. Briefly, graphene is a honeycomb-like crystal lattice of tightly packed sp^2^-bonded carbon atoms organized in a planar sheet, with excellent mechanical properties and superior thermal and electrical conductivity that make it a suitable candidate for a great number of applications [3,4,5,6,7]. Moreover, graphene is only a member of a larger class named ‘graphene-based materials’, which contains also oxidized, functionalized, modified graphene. All of these may now be prepared via bottom-up processes, such as epitaxial growth and CVD (e.g., classical graphene), as well as top-down methods, such as micromechanical or chemical cleaving/exfoliation of graphite or oxidized graphite. [8,9,10]. From these, in terms of yield at lower cost, the exfoliation of oxidized graphite is the most suitable one for obtaining 2D graphene materials due to its distinguishing characteristics: the use of graphite as an inexpensive raw material and the resulting graphene oxide (GO) as stable colloidal solutions [10,11,12].

Initially developed as a possible precursor for graphene, GO quickly gained popularity in the research community due to its scalable and low-cost synthesis [13]. Nowadays, GO is regarded as an unconventional 2D material that is easily functionalized and exhibits a behaviour similar to colloids, liquid crystals or polymers, depending on its environment, serving as a building block for a variety of microstructured materials, such as membranes or other nanoporous structures [14,15,16,17,18].

However, the chemical structure of GO has not been completely elucidated due to the multiple synthesis processes that produce different particularities of GO structure, which presents a variety of defects and oxygen ratios that affect the material’s reported capabilities [19]. Several structural models for GO have been proposed up to now [20,21,22]. The dynamic model proposed by Dimiev et al. further explained the origin of the acidity of GO solutions and the mechanisms altering the carbon framework during the synthesis process. They highlighted that the water molecules are responsible for transforming the structure, and that GO may not have a static structure [22]. Through both theoretical calculations and experimental investigations on multilayer GO samples, Kim et al. concluded that GO films undergo spontaneous alterations, with a relaxation time of roughly 35 days, when dispersed in water at room temperature [23]. These intriguing phenomena related to the meta-stability of GO in water led to the conclusion that the material is still undergoing spontaneous alterations due to its complex structure [24]. Knowing how the structure and surface properties change over time is important because it can reveal information about the types of chemical activities taking place on the surface and at the edges of GO nanosheets. Up to now, most of the theoretical studies performed in order to clarify the GO structure and its particularities have been focused primarily on single-layered sheets, neglecting multilayer films, or through classical molecular dynamics (MD) simulations, treating the oxygen functional groups as passive in water [25,26,27,28].

Our work aims to uncover and highlight the processes affecting the stability of GO dispersions in water through in-depth structural and surface property investigations carried out over a period of eight weeks after the synthesis process. The investigations were conducted on two fractions of multilayer, highly oxidized GO obtained by using an original modified Marcano-Tour process. These fractions were then used to develop GO samples weekly, resulting in two types of systems that were investigated: (i) GO nanosheets dispersed in water, for which the size distributions and ζ-potential were measured, and (ii) dried GO samples, for which structural analysis was performed. Based on the experimental results and the previously reported works discussed above, we successfully identified the two underlying mechanisms affecting the stability of GO dispersed in aqueous dispersions, which can be used to evaluate the variations of surface and structural properties of GO at different times after the synthesis. In this way, certain parameters (e.g., degree of oxidation, desired zeta potential, the amount of planar oxygen functionalities, etc.) can be tuned according to the desired application of the nanomaterial by using it at different times after the synthesis is finalised.

## 2. Materials and Methods

### 2.1. GO Synthesis through a Sono-Chemical Exfoliation Method

**Figure 1:**
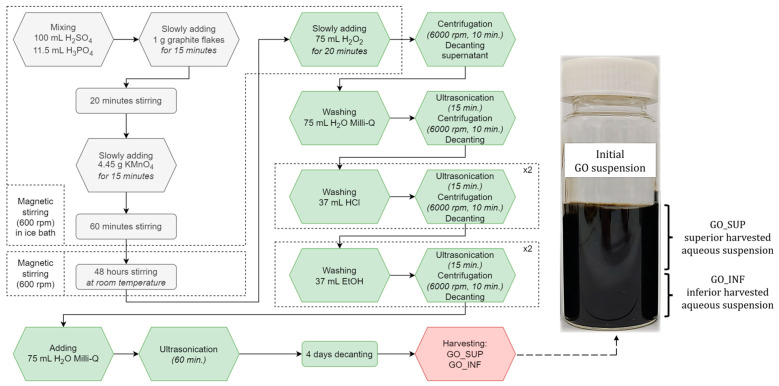
Schematic representation of the synthesis process of the obtained superior (GO_SUP) and inferior (GO_INF) GO aqueous suspensions.

The GO synthesis is based on the Marcano-Tour method’s primary raw reagents (i.e., graphite, H_2_SO_4_, H_3_PO_4_ and KMnO_4_) [29] with some additional steps that include sonicating, washing, centrifugation and decantation [30,31,32]. The synthesis pathway can be grouped in three steps and can be seen outlined in Figure 1. In the first step, mixing of H_2_SO_4_ (97%, SC Nordic Invest SRL, Cluj-Napoca, Romania) and H_3_PO_4_ (85%, Sigma Aldrich, St. Louis, MI, USA) in a 100:11.5 mL/mL ratio was realized. After 5 min, 1 g of graphite (99.9995%, powder, 100 mesh diameter, Alfa Aesar, Haverhill, MA, USA) was added in small portions as a controlled rain (i.e., a fifth of the total amount was added at first and after about 3 min the next fifth, and so on until all of the amount was added), under continuous mixture, for 20 min, in an ice bath. Then, 4.45 g KMnO_4_ (99%, Sigma Aldrich, St. Louis, MI, USA) were added as a controlled rain for 15 min under continuous stirring (magnetic stirring, 600 rpm), and the mixture was left for another 60 min under stirring in an ice bath. Afterwards, the ice bath was removed, and the mixture was left at room temperature for 4 days in ambient atmosphere.

In the second step, the pre-cooled mixture was stirred in an ice bath, and 75 mL H_2_O_2_ (3%, SC “Hipocrate 2000” SRL, Bucharest, Romania) were slowly added. In the ice bath, the mixture was stirred for another 20 min before being centrifuged for 10 min at 6000 rpm. The supernatant was decanted away. The solid was washed with 75 mL H_2_O Milli-Q (laboratory made), 37 mL HCl (35%, Sigma Aldrich, St. Louis, MI, USA) and 37 mL absolute ethanol (SC Nordic Invest SRL, Cluj-Napoca, Romania), in that order. After each washing, 15 min of ultrasonication, 10 min of centrifugation at 6000 rpm and decanting the supernatant were performed. Washings with HCl and absolute ethanol were repeated twice. The solid was mixed with 75 mL H_2_O Milli-Q after the last decantation, ultrasonicated for 60 min and kept in a sealed jar at room temperature for 4 days. The final step consisted of separating the GO aqueous suspension into two fractions with a harvesting syringe: GO_SUP (i.e., superior fraction of the jar, about 60 mL) and GO_INF (i.e., inferior fraction of the jar, about 20 mL). The two fractions were kept in separate jars. Weekly, two different systems were used to investigate the stability of the GO over time. Diluted water dispersions of the given fractions were prepared to be used for size distribution and ζ-potential measurements, and, similarly, each week, a given amount of material was dried on glass at room temperature for a week in order to be used for structural characterization.

### 2.2. Characterization Techniques

A Malvern Nano ZS90 Zetasizer particle analyser equipped with a He-Ne laser (633 nm, 5 mW) was used to examine particle size distributions and ζ-potential in a water colloidal solution of GO (0.25 mg/mL). Each investigation consisted of five sets of 30 measurements, all taken at a scattering angle of 90° and at a temperature of 25 °C. The laser attenuation level for each measurement was chosen automatically by the software.

Scanning electron microscopy (SEM) analysis was performed for dried GO samples with a Hitachi S-4700 Type II cold held emission instrument equipped with a Rontec QX2-EDX spectrometer.

Raman spectra were recorded by using a Renishaw InVia Reflex Raman spectrometer equipped with an air-cooled RenCam CCD detector. A 532 nm laser line with a power of 200 mW was employed as the excitation source. For recording the spectra, a 30 s exposure time at 0.1 percent power was used. The spectral resolution was 4 cm^−1^.

FT-IR absorption spectra were recorded with a JASCO 6600 spectrometer (IR, Jasco, Tokyo, Japan) within the range of 400–4000 cm^−1^, at room temperature, with a spectral resolution of 4 cm^−1^, using the KBr pellet technique.

XRD diffractograms were obtained by using a Shimadzu XRD-6000 diffractometer (Kyoto, Japan), operating with CuK_α_ radiation (λ = 1.54 nm) and a Ni filter. The diffraction patterns were recorded in the 2θ range between 3° and 80° with a scan speed of 2°/min.

The XPS spectra were recorded with a SPECS PHOIBOS 150 MCD system employing a monochromatic Al-Kα source (1486.6 eV), a hemispherical analyser and a charge neutralization device. Samples were fixed on double-sided carbon tape and care was taken to ensure that the sample particles covered the tape. Experiments were performed by operating the X-ray source with a power of 200 W, while the pressure in the analyse chamber was in the range of 10-9–10-10 mbar. The binding energy scale was charge referenced to the C1s at 284.6 eV. Elemental composition was determined from survey spectra acquired at pass energy of 60 eV. High resolution spectra were obtained using analyser pass energy of 20 eV. Analysis of the data was carried out with Casa XPS software. A Shirley background was used for all curve-fitting, along with the Gaussian/Lorentzian product form (70% Gaussian and 30% Lorentzian).

## 3. Results

A sono-oxidative exfoliation of graphite was carried out to obtain GO aqueous solutions. This three-step synthesis pathway was successfully used in previous studies to obtain self-assembled [30] and drop-casted-dried-exfoliated GO membranes [31,32]. In the present study, in the final step of the synthesis, the obtained GO material was dispersed in water and kept for 4 days unperturbed in ambient conditions. In this stable suspension, the GO nanosheets with different size and weight were placed at a certain aqueous suspension level, where buoyant forces are balanced with the gravitational force acting on them. Moreover, this balance is tightly related to the density of the functional groups (i.e., hydroxyl, carbonyl, carboxyl and epoxy) that populate both the planes and the edges of the GO nanosheets. This provides the future properties that can be exploited in further specific applications (e.g., semiconductors, sensors, functionalization platforms etc. [10,11,14,17]).

In order to investigate the structural differences and their evolution in time between the GO nanosheets with a high level of buoyant points and those that are placed at a lower level of the suspension jar, two types of GO suspensions were harvested and analysed: GO_SUP (i.e., superior fraction of the jar) and GO_INF (i.e., inferior fraction of the jar). Moreover, the differences related to the exfoliation, oxidation degree and recombination tendency for both fractions were evidenced.

The experimental results obtained for both GO suspensions were evaluated to determine a time evolution of the surface and structural properties. The results are presented as a dependence of the sought-after parameters on time.

### 3.1. Scanning Electron Microscopy (SEM)

**Figure 2:**
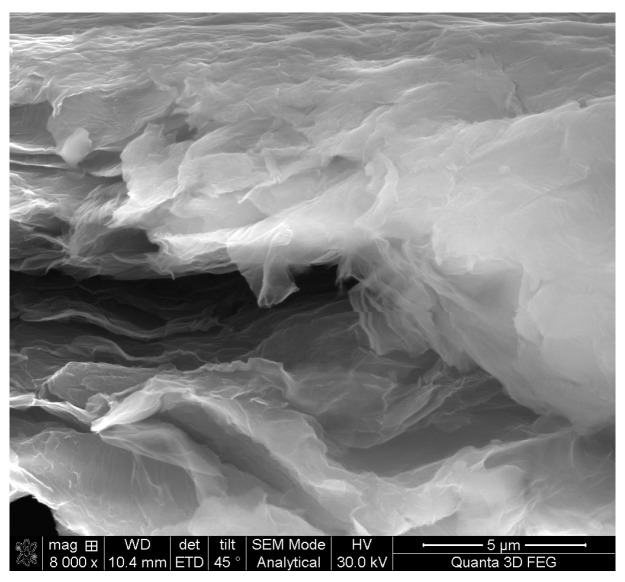
SEM image of the edge of a self-assembled GO film showing the wrinkled nanosheets at the edge of the microstructure.

In order to visualize the nanosheet features of the samples, SEM investigation was performed on dried GO materials. Figure 2 presents an image of the edge of a formed GO film. A disturbed, multi-layer, stacked structure that can be transparent in some zones, where the number of nanolayers is low, can be identified. The GO nanosheets are wrinkled, with an irregular geometry, and form 3D micrometric-sized structural entities (i.e., multi-micrometric area sheets). Through EDX analysis of the visualized zone, a carbon to oxygen atomic ratio (C/O) value of about 2:1 was obtained.

### 3.2. Dynamic Light Scattering (DLS) and Zeta Potential (ζ-Potential)

**Figure 3:**
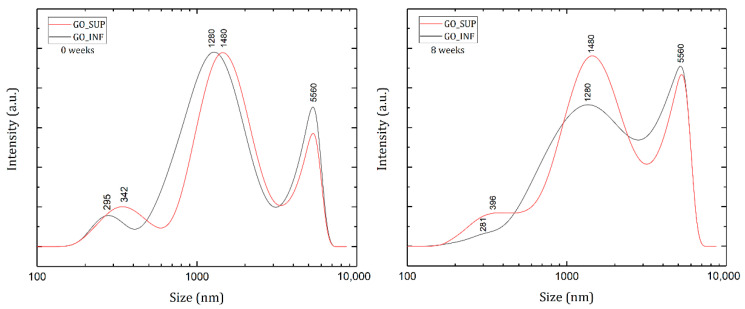
Size distributions by intensity obtained for GO suspension fractions GO_SUP and GO_INF immediately after the synthesis process was done (**left**) and 8 weeks later (**right**).

The hydrodynamic diameter values of the GO nanosheets dispersed in water were obtained by DLS measurements immediately after completing the synthesis process and 8 weeks later, and they are represented in Figure 3. The results show a size distribution consisting of three peaks: a small peak at 300–400 nm, a second peak as the highest at 1280–1480 nm and a third peak with an intermediate intensity at 5560 nm.

As DLS data are completely relevant only to 3D spherical structures, the data acquired can only be qualitatively discussed in order to estimate the size variation in time of the 2D GO nanosheets by comparison between different results at different points in time. After eight weeks, an increase of the intensity corresponding to the third peak and the region between it and the second peak is observed, which indicates a slight aggregation of the GO nanosheets in the large size domain. This corresponds to a relatively increased stability of the dispersion against aggregation. It is worth noting that the origin of the peaks is difficult to interpret; the first two peaks may appear due to the planar structure of the GO nanosheets, for which a strong anisotropy of the diffusion coefficients (parallel and perpendicular motion) may be the cause [33]. The third peak may be the result of some aggregated GO sheets or others that might not be completely exfoliated after the synthesis. It has consistently appeared at the same corresponding size (5560 nm), possibly due to the instrument limitations on displaying results on such a large-sized domain for a multimodal distribution [34,35].

**Figure 4:**
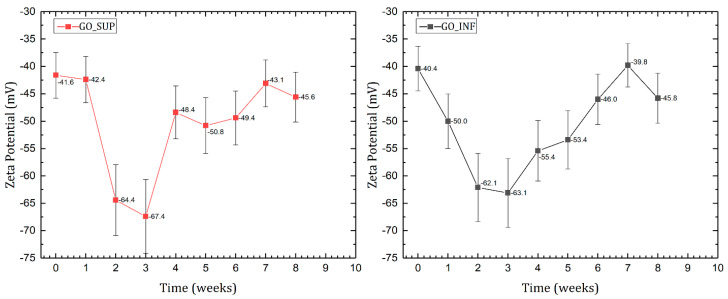
ζ−potential variation for GO suspension fractions GO_SUP (**right**) and GO_INF (**left**) over the course of 8 weeks after the synthesis process.

The mean ζ-potential variation in time for the two fractions is displayed in Figure 4. A significant decrease can be observed in the first three weeks, from values around −40 mV to a maximum negative value of −67 mV for GO_SUP and −63 mV for GO_INF, indicating an increase of the number of negative surface charges in the electrical double layer. Afterwards, the mean ζ-potential returns to values around −45 mV, with slight variations. This corresponds to a high stability of the dispersion of GO nanosheets, which can also be seen in the slight aggregation observed after the DLS measurements over the eight weeks due to the strong electrostatic repulsion between them. These results are the first indication of the processes taking place at the surface of the GO nanosheets affecting the surface charge distribution. 

### 3.3. Raman Spectroscopy

**Figure 5:**
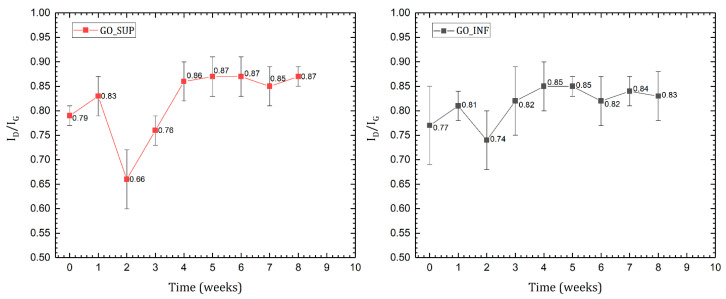
The time evolution of the D and G band intensity ratio from the Raman spectra of dried GO_SUP (**left**) and GO_INF (**right**) samples.

**Figure 6:**
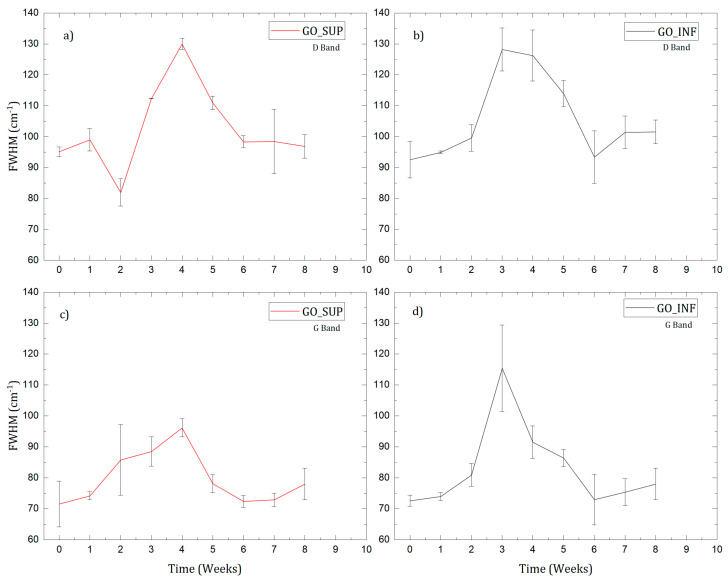
The FWHM values obtained after processing the D (**a**,**b**) and G bands (**c**,**d**) from the Raman spectra of dried GO_SUP and GO_INF samples.

The Raman spectra of the dried GO_SUP and GO_INF samples were recorded weekly. Each spectrum exhibits the typical features of carbon materials, with the presence of the G-band and D-band, a notable aspect being the absence of the 2D band, which is a first clue to the highly oxidized character of the as-prepared GO structures [36,37,38]. By fitting the spectra, the intensity ratios of the D and G bands and their FWHM were obtained, and they are illustrated as a function of time in Figure 5 and Figure 6, respectively. The intensity ratio $I_{D}/I_{G}$ is commonly used to estimate the degree of oxidation for the GO structures, which is proportional to the average distance between the structural defects on the sp^2^ carbon basal plane [39,40,41]. It was concluded that for GO structures with a low degree of oxidation (i.e., a lower density of defects as GO basal plane holes and/or the functional groups, such as carboxyl carbonyl, hydroxyl, epoxy), an increase of the $I_{D}/I_{G}$ ratio corresponds to an increase in the degree of oxidation, whereas for GO structures with a high degree of oxidation, a decrease of the $I_{D}/I_{G}$ ratio corresponds to an increase of the oxidation degree, as the structural defects overwhelm the pristine carbon lattice [39,41]. To correctly interpret the experimental data and identify the correct behaviour of the $I_{D}/I_{G}$ ratio when increasing/decreasing the degree of oxidation, the widths of the D and G bands must also be taken into consideration. The absence of the 2D band and its overtones and the large FWHM values of the D and G bands are an indication of the highly oxidized region of the obtained GO structures [39,42,43]. Thus, the decrease of the $I_{D}/I_{G}$ ratio observed for GO_SUP and GO_INF in the first weeks can be attributed to an increase in the density of defects on the carbon basal plane. Afterwards, we observe the $I_{D}/I_{G}$ ratio returning to values around 0.85, indicating a decrease in the oxidation degree, which is also supported by the decrease of the FWHM of the D and G bands to values close to the initial ones after the synthesis.

### 3.4. Fourier Transform IR Spectroscopy (FT-IR)

**Figure 7:**
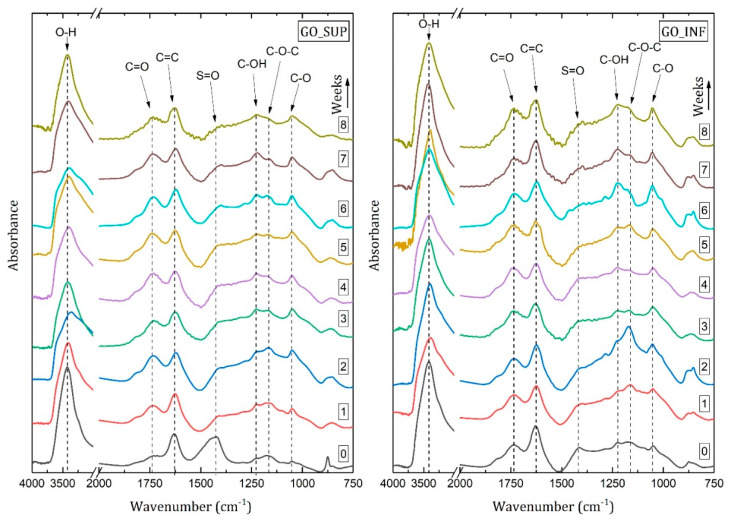
FT−IR spectra recorded weekly over the considered time period for the dried GO_SUP (**left**) and GO_INF (**right**) samples.

FT-IR measurements were performed weekly on dried GO samples. Figure 7 displays a successive representation of the IR spectra obtained from the dried GO_SUP and GO_INF samples in the eight-week time frame. The absorption bands were identified and assigned according to the existing literature [19,44,45,46]. Thus, the absorption band around 3420 cm^−1^ is due to the O-H stretching vibration, the band around 1735 cm^−1^ is given by the C=O stretching vibration, the signal around 1640 cm^−1^ is attributed to the stretching vibration of the C=C bonds and the absorption band around 1430 cm^−1^ is assigned to the S=O stretching vibration [47]. The signals recorded in the 1250–1000 cm^−1^ wavenumber region are due to the stretching vibrations of the C-OH (around 1220 cm^−1^), C-O-C (around 1165 cm^−1^) and C-O bonds (around 1050 cm^−1^) [19,44,45,46]. 

**Figure 8:**
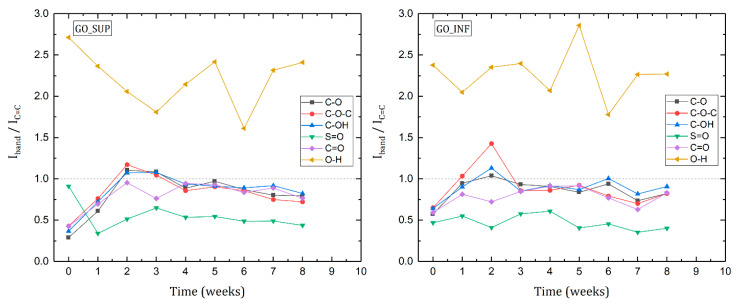
The intensity ratio of the functional group absorption bands and the 1640 cm^−1^ reference band for the dried GO_SUP (**left**) and GO_INF (**right**) samples over a time period of eight weeks after synthesis.

To observe the time evolution of the functional groups attached to the basal planes, the ratio of the intensities of each absorption band corresponding to a functional group vibration to the intensity of the 1640 cm^−1^ band has been evaluated and is represented in Figure 8 for GO_SUP and GO_INF. The absorption band from 1640 cm^−1^ that is due to the stretching vibrational mode of the C=C bonds has been chosen as a reference band because there are no significant variations of its intensity in the eight-week time frame. 

For both types of samples, a similar trend is observed, with an increase of the intensity of the absorption bands corresponding to the oxygen functional groups, relative to the reference band for the first two weeks and a slow decrease afterwards. However, an important difference between the two fractions can be observed at the beginning of the evaluation period, with an increased intensity of the absorption band due to S=O vibration, relative to the reference one for the GO_SUP immediately after the synthesis process is finished. 

### 3.5. X-ray Diffraction (XRD)

**Figure 9:**
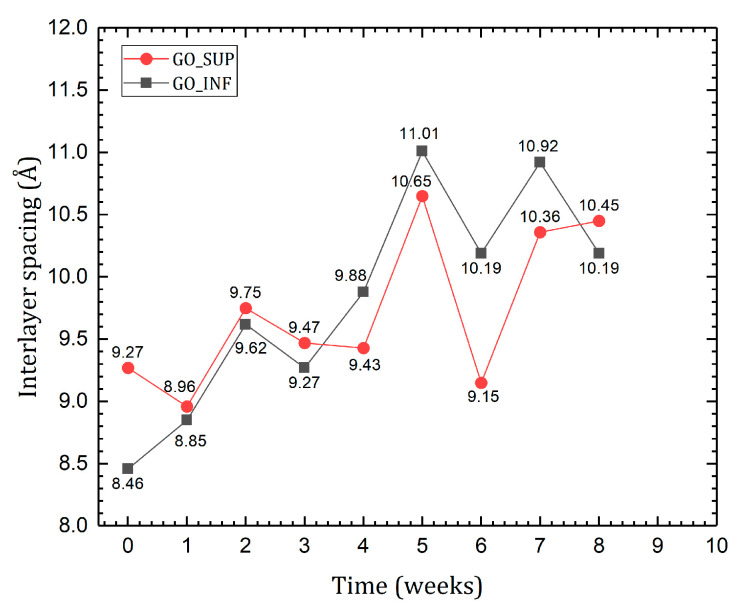
Calculated interlayer distance variation of the GO sheets over the course of eight weeks after the synthesis.

XRD patterns obtained for the dried GO samples in atmospheric conditions feature a sharp peak at values between 8.29° and 9.85° for GO_SUP and between 8.02° and 10.52° for GO_INF. The interlayer spacing was calculated using Bragg’s diffraction law, and its time variation is represented in Figure 9. Small variations for GO_SUP and a slow increase for GO_INF can be observed for the first four weeks. Afterwards, large variations and a similar behaviour can be noticed for both of the GO fractions.

### 3.6. X-ray Photoelectron Spectroscopy (XPS)

**Figure 10:**
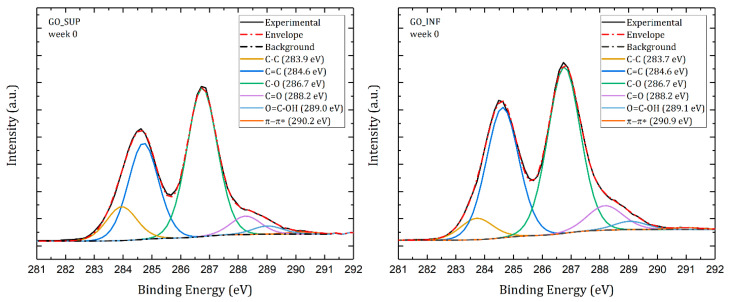
Deconvoluted C1s core-level XPS spectra of the dried GO_SUP (**left**) and GO_INF (**right**) samples obtained immediately after synthesis.

High-resolution XPS measurements on the C1s core level were carried out in order to gain additional knowledge about the surface modifications of the dried GO sheets in the first four weeks. In this respect, deconvolutions of the XPS spectra were performed for all investigated samples, similar to the case illustrated in Figure 10.

The following bond types are typically represented in a GO material’s C1s core-level spectrum: C-C (sp^3^ carbon, around 283.8 eV), C=C (sp^2^ carbon, around 284.6 eV), C-O (hydroxyl or epoxy, around 286.7 eV), C=O (carbonyl, around 288.2 eV) and O=C-OH (carboxyl, around 289.1 eV) [48,49]. Graphene materials frequently exhibit the π-π* satellite peak at about 290 eV, which denotes a more orderly chemical environment, a characteristic of aromatic/conjugated systems.

**Figure 11:**
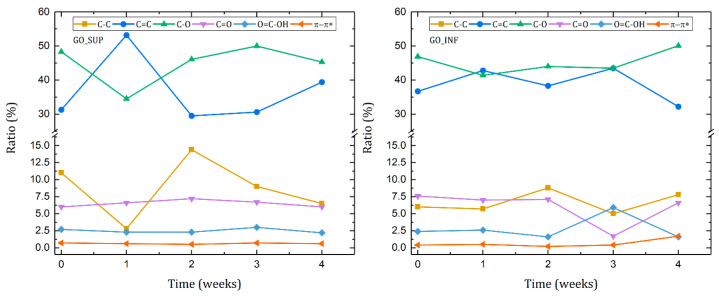
Peak ratios of the deconvoluted C1s core-level spectra of the dried GO_SUP (**left**) and GO_INF (**right**) samples over the course of the four weeks after the synthesis.

The evolution of the proportions of each bond type identified in the deconvolutions of the XPS spectra for the four weeks taken into account is represented in Figure 11. For both types of GO fractions, we can observe a higher amount of C=C and C-O functional groups, indicating that a large proportion of the material is composed of graphenic domains and centre-surface functional groups, such as epoxides or hydroxyl groups. By comparison, a lower ratio of C-C domains and edge functional groups, corresponding to C=O and O=C-OH bonds, is observed for both samples. We can also notice that the ratio of π-π* is low, which indicates that the structure of the GO samples is highly disordered.

It is important to note that the analysis was carried out on dried GO samples, implying that the number of edge functional groups observed after drying and assembly is significantly lower than in GO samples dispersed in water. After drying the samples, we can assume that the observed amounts of centre-surface functional groups correspond to trapped O and H atoms between the planes, whereas the edge functional groups may correspond to carbonyl and carboxylic groups at the edges of intra-planar defects, such as holes or vacancies. 

Weekly variations in both GO fractions can still be seen, with larger changes in the dried GO_SUP fractions. Edge functional group ratios (C=O and O=C-OH) are relatively constant for both GO samples, with the exception of the third week for GO_INF, where the carbonyl and carboxylic amounts change abruptly before returning to similar values in the fourth week. The π-π* interactions for GO_INF become noticeable only in the fourth week, indicating a brief increase in the order of the chemical environment.

The proportions of sp^2^ and sp^3^ carbon (C=C and C-C, respectively) and the amount of centre-surface functional groups (C-O) fluctuate the most. In both GO samples, we first see an increase of the ratio of graphitic sp^2^ carbon (C=C), while the amount of sp^3^ carbon (C-C) and centre-surface functional groups (C-O) decreases. However, we see a maximum amount of sp^3^ carbon (C-C) and a large amount of centre-surface oxygen functional groups (C-O) in the second week, indicating that around this time, the carbon planes become highly oxidized. In the case of GO SUP, a restoration of the sp^2^ carbon planes (C=C) appears to occur after the second week, with an associated decrease in sp^3^ carbon (C-C) and centre-surface oxygen functional groups (C-O). The same process is not noticeable in the case of GO INF, which has a decrease in sp^2^ carbon (C=C) and an increase in sp^3^ carbon (C-C) and centre-surface oxygen functional groups (C-O) after four weeks.

**Figure 12:**
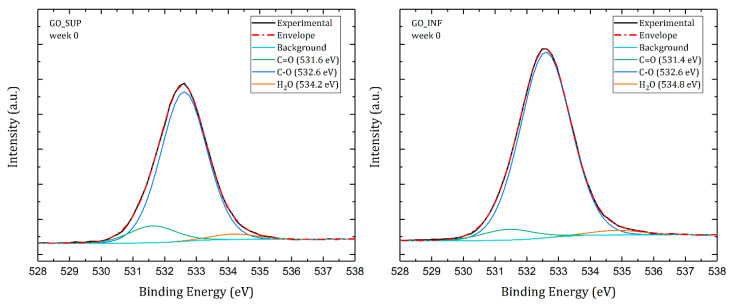
Deconvoluted O1s core-level XPS spectra of the dried GO_SUP (**left**) and GO_INF (**right**) samples obtained immediately after synthesis.

**Figure 13:**
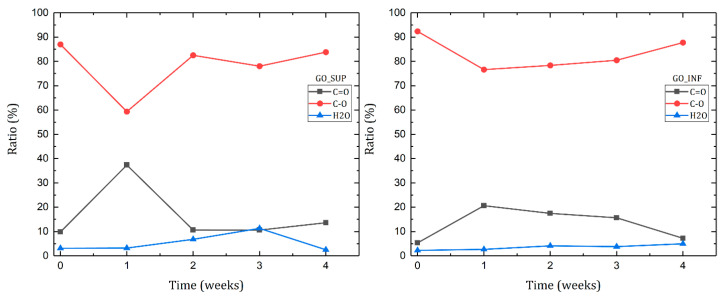
Evolution of the ratios of each bond type identified in the deconvoluted O1s core-level spectra of the dried GO_SUP (**left**) and GO_INF (**right**) samples over the course of the four weeks after the synthesis.

Further research was carried out by analysing the deconvoluted peaks of the O1s core level (Figure 12). Deconvolution of the O1s core-level spectra typically yields two peaks: oxygen doubly bonded to aromatic carbon (C=O, at around 531 eV), oxygen single bonded to aliphatic carbon (C-O, at around 532 eV) and an additional peak assigned to chemisorbed or intercalated adsorbed water molecules (H_2_O, at around 534 eV) [50,51]. Figure 13 depicts the weekly changes that were recorded. The variations of the C=O and C-O peaks are strikingly similar to those observed for the same peaks obtained through deconvolution of the C1s core-level spectra. Surprisingly, it is also discovered that the amount of intercalated water molecules increases weekly.

## 4. Discussion

### 4.1. Correlation of Experimental Observations

These results, taken together, indicate a meta-stability of both GO fractions. The results obtained for the GO dispersions in water (DLS and ζ-potential measurements) appear to be in accordance with one another. A qualitative observation of the size distributions shows small changes that can be attributed to particle aggregation over the course of eight weeks. This observation is sustained by the large negative values for the ζ-potential, indicating strong electrostatic repulsion between the GO nanosheets due to the large negative surface charges in the electrical double layer. The aggregation occurs due to the van der Waals interactions between the functional groups present at the surface.

The Raman, FT-IR, XPS and XRD methods offer valuable information regarding the structural and surface changes on the GO nanosheets in the eight-week time frame. Taken together, we see that the $I_{D}/I_{G}$ ratio decreases in the second week (greater changes in GO_SUP and smaller changes in GO_INF), while the FWHM of the D and G bands grows, corresponding to the structural functionalization that affects the carbon planes. Taking this into account, as well as the absence of the 2D band and any overtone in the Raman spectra, we can conclude that the obtained GO structures are highly oxidized. This result is also confirmed by the deconvoluted XPS spectra, for which we can observe a predominantly higher intensity of the C-O peak as compared to the rest. This is further supported by the evolution of the IR absorption bands of the oxygen functional groups, which increase in the first three weeks. Note the rapid increase in intensity of the centre-surface oxygen functionalities bands given by the C-O-C, C-OH and C-O group vibrations relative to the intensity of the reference band corresponding to the C=C bond stretching vibration. In the same time interval of the first 3–4 weeks, the interlayer spacing suffers small variations or grows for GO_SUP and GO_INF, respectively. This corresponds to the formation of oxygen functional groups by oxidating the structure, replacing the sulfur ones existent after the synthesis in the case of GO_SUP. In the case of GO_INF, the oxygen functional groups add up to the already present sulfur groups in the interlayer spacing. The information obtained from the deconvoluted XPS spectra also suggests that the majority of the changes in the first four weeks occurs on the carbon planes, with the peaks corresponding to the edge functional groups, such as carbonyl and carboxyl, undergoing few changes, while the largest variations occur to the centre-surface oxygen functionalities and their subsequent effect on the sp^2^ carbon planes.

Knowing that in the IR spectra of GO, the O-H absorption band is due to the vibration of the -OH groups in the tertiary alcohols and, to a greater extent, to the vibration of -OH groups from H_2_O molecules adsorbed or intercalated between the planes, we expect to see a proportional dependence of the intensity of this band on the interlayer distance, caused by the presence of the large number of H_2_O molecules between the carbon layers [21,22,30]. However, in the first four weeks, the interlayer distance grows for both fractions, while the intensity of the O-H absorption band decreases (for GO_SUP) or suffers small variations (for GO_INF). After four weeks, though, there appears to be a correlation between the interlayer distance and the intensity of the O-H absorption band. This can be seen easily if the interlayer distance values in the 1st and 5th week are compared for GO_SUP, as the corresponding intensity ratios of the O-H absorption band to the C=C band have similar values. The same comparison can be made for the 2nd and 7th weeks for GO_INF. This observation is also confirmed by the XPS spectra, as we see that the amount of intercalated H_2_O molecules increases weekly, starting from a low amount of 3.14% and 2.25% for GO_SUP and GO_INF, respectively. This suggests that in the first four weeks, the origin of the O-H stretching band in the IR spectra is due to the presence of the -OH groups in the tertiary alcohols, rather than due to the vibration of the -OH groups from the H_2_O molecules intercalated, which seem to keep forming and whose influence might be dominating after the first four weeks.

The information gathered through the analysis of the two systems—GO nanosheets dispersed in water and dried GO samples—seems to be in mutual accordance. The enhancement of the IR absorption bands corresponding to the oxygen functional groups and the XPS ratios observed for the sp^2^ (C=C), sp^3^ carbon (C-C) and centre-surface functional groups (C-O) in the 2nd and 3rd weeks confirm the decrease of the ζ-potential, as it is known that oxygen functional groups are responsible for the negative surface charge [52,53]. Furthermore, the increase of the ζ-potential values starting with the third week might be correlated with the decrease of the intensities of the IR bands assigned to the surface functionalities vibrations, while the functional groups are being replaced by intercalated/adsorbed water molecules supported by the deconvolution of the O1s core-level spectra, which show that the number of intercalated H_2_O molecules is increasing.

### 4.2. Mechanisms Underlying the Meta-Stability of GO in Aqueous Dispersions

The observed changes highlight the meta-stability of the synthesized GO fractions when dispersed in water. There are two possible mechanisms that can describe the structural and surface changes in time, which can be listed as follows, based on the observed structural changes:

- The formation of oxygen-based functional groups at the surface of the GO nanosheets, through the interaction with the water molecules [22,30]. This process seems dominant during the first three to four weeks and is confirmed by the following observations: the enhancement of the IR absorption bands corresponding to the identified functional groups vibrations; the decrease of sp^2^ carbon (C=C) and the increase of the amount of centre-surface bonds (C-O) and sp^3^ carbon (C-C) in the XPS spectra; the independent evolution of the interlayer spacing of the O-H absorption band intensity, which indicates the lack of adsorbed H_2_O molecules in the interlayer space; an increase in the oxidation degree; and the decrease of the ζ-potential value.
- The partial restoration of the carbon planes, through the transformation of the existing functional groups into H_2_O molecules adsorbed on the surface [23]. The process is observed after the first three or four weeks, but its presence earlier might be hidden by the first mechanism, as confirmed by the XPS spectra. It is supported by the following experimental observations: the decrease of the intensities of the IR absorption bands corresponding to the oxygen functional groups vibrations; the slow increase in the amount of H_2_O molecules in the XPS spectra in the first four weeks; the strong correlation between the interlayer spacing and the intensity of the O-H absorption band, indicating the presence of adsorbed H_2_O molecules in the interlayer spacing; a decrease in the oxidation degree; and an increase of the ζ-potential.

The two mechanisms have been studied theoretically earlier, with the first being described by Dimiev et al. in their structural model as the cleavage of carbon bonds through the nucleophilic attack of H_2_O molecules on a H atom in a hydroxyl group [22]. The process also releases a H_3_O^+^ cation, responsible for the acidity of GO solutions. Still, this model does not account for any interactions that may appear between the functional groups present at the surface [24,30]. These types of interactions have been studied by Kim et al. through DFT calculations and XPS experimental investigations on multilayer GO samples obtained through the oxidation of epitaxial graphene films grown on a SiC wafer via a Hummers synthesis method, for which it was concluded that the formation of adsorbed water molecules on the planes is energetically favourable [23]. Although in this work an original modified Marcano-Tour synthesis method has been used for the sample preparation, we must admit that the fundamental processes that take place are identical [30].

The two identified mechanisms successfully explain the observed structural and surface changes over the course of eight weeks after the synthesis and can form the basis of a dynamic structural model of GO in aqueous dispersions. However, it must be noted that this is a phenomenological model that does not offer a complete answer about the stability of GO structures dispersed in water. Due to the complexity (i.e., variety in size/area and edge features) of the GO structure and the interactions between the numerous oxygen-based functional groups on the surface, there may not be a specific moment in time when the system becomes stable. Moreover, because of the negative ζ-potential observed, the interactions between the GO and H_2_O molecules happen relatively slowly, as their access in the proximity of the carbon planes is blocked by the large number of ions in the electrical double layer. From a different point of view, however, this may be considered an advantage, as it grants us the possibility to observe the processes that happen at the surface. It might be that for GO structures with a higher ζ-potential, the processes might take less time to happen due to the increased accessibility of the H_2_O molecules in the proximity of the carbon planes. Nonetheless, understanding the two mechanisms that influence the structural and surface properties observed for GO dispersions in aqueous solutions allows us to identify the best time after synthesis to use them in targeted applications requiring, for example, a lower ζ-potential or a greater amount of planar oxygen functionalities. This way, it is possible to improve some structural and surface properties without changing the synthesis pathway.

## 5. Conclusions

In the present article, two fractions of GO synthesized by an original modified Marcano-Tour method were obtained and investigated over an eight-week period in two different systems: GO nanosheets dispersed in water (as a solvent) and dried GO samples. Through the use of DLS, zeta potential measurements, XRD, XPS, Raman and FT-IR Spectroscopy, the alteration of the structural and edge-surface properties of 2D GO nanosheets was monitored. The experimental methods employed allowed for a complete characterization of both GO fractions, for which no major difference has been found besides the higher amount of sulfur functionalities of GO_SUP right after the synthesis. Moreover, the two fractions followed the same evolution pattern in the time frame considered. The correlation of each obtained results allowed for the identification of two mechanisms that take part in the dynamic evolution of the system considered, which were found to be in agreement with two previously published theoretical models, providing experimental confirmation.

Taken together, the two mechanisms successfully describe the variations in the structural and surface properties of GO dispersed in aqueous solutions. After the synthesis is finished, the cleavage of carbon bonds at the formation of oxygen-based functional groups continues in the presence of water, while the formation of adsorbed water molecules as a result of the system’s energy equilibration becomes visible after 3–4 weeks, after the first mechanism stops. The processes were revealed due to the highly negative ζ-potential of the GO nanosheets, which limits the access of H_2_O molecules in the proximity of the carbon planes due to the great number of ions in the electrical double layer. As such, it might be possible that for GO structures that possess a lower surface charge, the mechanisms would not be easily observed. 

The synthesized GO fractions prove to be promising platforms for functionalization due to their excellent surface characteristics. The two identified mechanisms are useful in determining the evolution in time of different structural and surface parameters, allowing for targeting specific properties, depending on the desired application, by using the synthesised nanomaterial at different times after the synthesis.
